# An Unusual Cause of Precordial Chest Pain

**DOI:** 10.1155/2013/342096

**Published:** 2013-01-10

**Authors:** Sevket Ozkaya, Kamil Furtun, Canan Yuksel, Adem Dirican, Serhat Findik

**Affiliations:** ^1^Department of Pulmonary Medicine, Samsun Medical Park Hospital, Samsun, Turkey; ^2^Department of Pulmonary Medicine, Faculty of Medicine, Rize University, Rize, Turkey; ^3^Department of Thoracic Surgeon, Samsun Chest Diseases and Thoracic Surgery Hospital, Samsun, Turkey; ^4^Samsun Pathology and Cytology Centre, Samsun, Turkey; ^5^Department of Pulmonary Medicine, Faculty of Medicine, Ondokuz Mayis University, Atakum, Samsun, Turkey

## Abstract

Extraskeletal chondrosarcoma in anterior mediastinum is very rare. A 45-year-old male patient was admitted to the hospital with precordial chest pain. A large and well-shaped mass in the anterior mediastinum was seen radiologically, and there was a clearly compression of the heart by the mass. The lesion was totally resected, and extraskeletal mediastinal chondrosarcoma was histopathologically diagnosed. We aimed to present and discuss the radiologic, clinic, and histopathologic features of unusual presentation of extraskeletal chondrosarcoma in a case.

## 1. Background

Chondrosarcoma is a malignant mesenchymal tumor arising from cartilage-forming tissues involving the bones (bone chondrosarcoma) or the soft tissues (extraskeletal chondrosarcoma). Bone chondrosarcoma usually affects middle-aged to elderly adults, and the pelvic bones, ribs, shoulder girdle, and long bones are the most common locations. Extraskeletal chondrosarcoma usually affects the deep soft tissues of the extremities and the region of head and neck [1, 2]. Extraskeletal chondrosarcoma in anterior mediastinum is very rare. We present a patient with extraskeletal chondrosarcoma in anterior mediastinum, which was located on the heart.

## 2. Case Presentation

**Figure 1:**
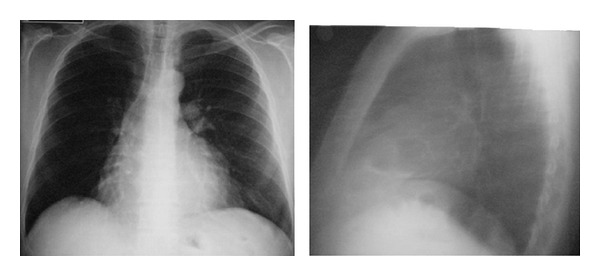
Posteroanterior and lateral chest radiography showing a shadow which superimposed on the shadow of his heart.

**Figure 2:**
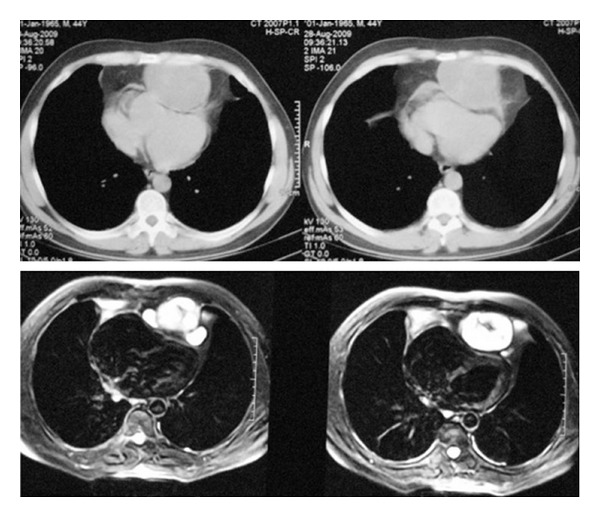
Thorax CT and MRI showing the large, encapsulated, well-defined anterior mediastinal mass lesion. The mass lesion was obviously compressing the heart.

A 45-year-old, nonsmoker male was admitted to the hospital with precordial chest pain. Blood tests results were normal. Chest roentgenogram was initially thought to be normal. But a masslike opacity was seen on the heart (Figure 1). Thorax computed tomography (CT) and magnetic resonance (MR) imaging demonstrated a large and well-shaped mass in the anterior mediastinum and clearly compression of the heart by the mass (Figure 2). 

**Figure 3:**
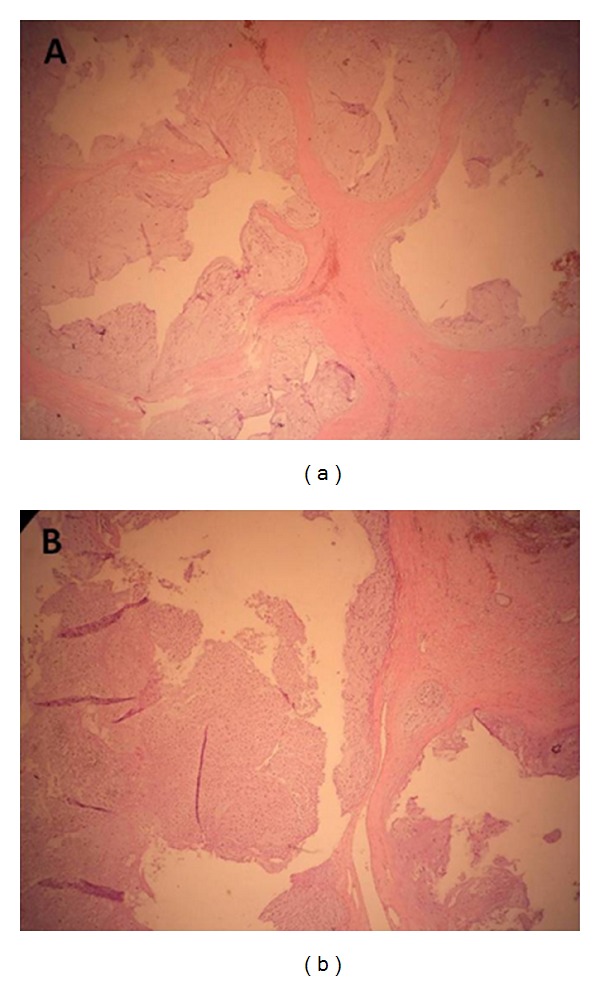
Photomicrograph of the resected tumor showing (Hematoxylin and Eosin, ×100) the well-differentiated chondrosarcoma with chondrocyte atypias and neoplastic chondrocytes.

**Figure 4:**
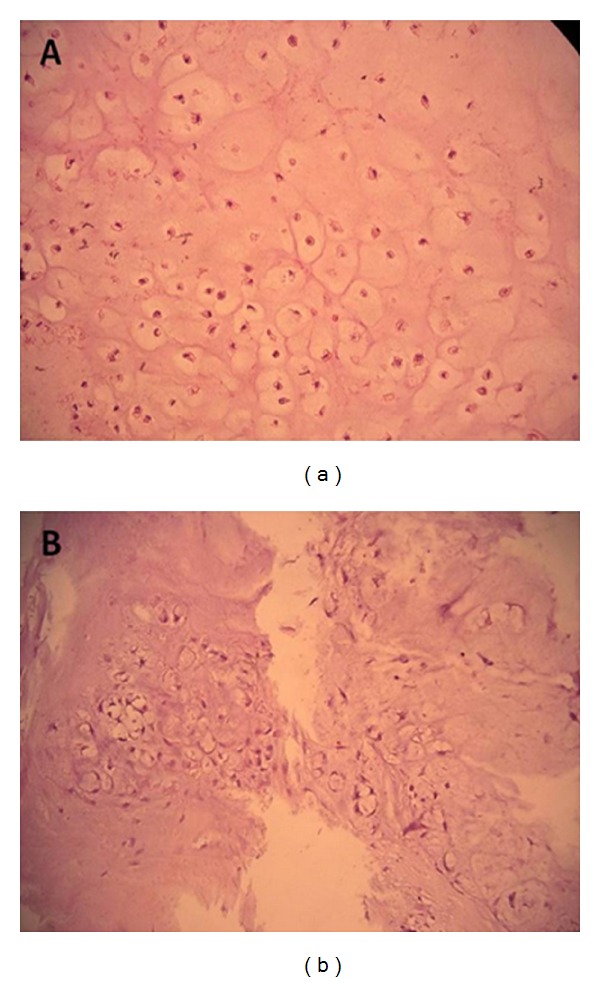
Photomicrograph of the resected tumor showing (Hematoxylin and Eosin, ×400) increased cellularity, marked atypia, and pleomorphism; mitosis.

Pulmonary function tests revealed normal spirometric values. The fiberoptic bronchoscopy was performed, and it was normal. Whole-body PET-CT with low-dose CT protocol was performed before the surgery, and there was no F18-fluorodeoxyglucose (FDG) uptake in lesion or any part of body. The surgical excision was planned. There was no relationship between the mass and adjacent structures. An encapsulated mass was totally removed. Pathological examination revealed a well-differentiated chondrosarcoma with chondrocyte atypias and neoplastic chondrocytes (Figures 3 and 4). No recurrence was seen in the 2-year followup. The approval of patient and institution were taken to use their records for our study. Written informed consent was obtained from the patient for publication of this case report and accompanying images.

## 3. Conclusions

Chondrosarcomas are presumably derived from primitive precartilaginous mesenchymal cells [3]. It may occur in extraskeletal locations and mainly in the soft tissues of the orbit, the cranial and spinal meningeal coverings, and lower limbs [4]. Primary chondrosarcoma of the anterior mediastinum without any continuity with cartilage-containing organs are extremely rare. In the present case, chondrosarcoma was located on anterior mediastinum and extraskeletal. 

Chondrosarcoma is more frequently found in patients from the fourth to seventh decades, with a male predominance [2, 5]. Local pain is the most frequent presenting symptom of patients with this neoplasm [1, 6, 7]. Precordial chest pain was main symptom in our case. Also, the mass lesion was marked compressing on the heart.

Chest radiography, thorax CT, and MRI depicts lobulated, well-shaped soft tissue mass, as in our case. Mediastinal chondrosarcomas also shares imaging findings with other skeletal and soft-tissue tumors. Brenner et al. reported that the combination of pSUV on PET imaging and histopathologic tumor grading in chondrosarcoma might be helpful for determining a more accurate diagnosis [8]. Histopathologic examination shows chondrocyte atypias and neoplastic chondrocytes, as seen in our case. 

The wide local excision is recommended for treatment. In the present case, mediastinal chondrosarcoma was totally resected. The patient did not receive radiotherapy because all the surgical margins were tumor-free. According to reported one article, the primary chondrosarcomas of anterior mediastinum may have a less aggressive clinical course than previously recognized [3]. There is no recurrence during two years after surgery. Burt et al. reported that the 47 primary sarcomas of the mediastinum and mediastinal chondrosarcoma was seen in only one patient. Also, factors significantly affecting survival were tumor grade and radicality of resection [9]. 

In conclusion, the extraskeletal chondrosarcoma in anterior mediastinum is very rare and usually overlooked on chest radiography. The complete surgical excision is enough for treatment, and prognosis is better than other chondrosarcomas.
